# Diagnostic Interpretation Guidance for Pediatric Enteric Pathogens: A Modified Delphi Consensus Process

**DOI:** 10.1155/2018/2589826

**Published:** 2018-09-27

**Authors:** Antonia S. Stang, Melanie Trudeau, Otto G. Vanderkooi, Bonita E. Lee, Linda Chui, Xiao-Li Pang, Vanessa Allen, Carey-Ann D. Burnham, David M. Goldfarb, Judy MacDonald, Brendon Parsons, Astrid Petrich, Frank Pollari, Phillip I. Tarr, Graham Tipples, Ran Zhuo, Stephen B. Freedman

**Affiliations:** ^1^Departments of Pediatrics, Emergency Medicine and Community Health Sciences, University of Calgary, Alberta Children's Hospital Research Institute, Calgary, Alberta, Canada; ^2^Department of Pediatrics, University of Calgary, Calgary, Alberta, Canada; ^3^Departments of Pediatrics, Microbiology, Immunology and Infectious Diseases, Pathology & Laboratory Medicine and Community Health Sciences, University of Calgary, Alberta Children's Hospital Research Institute, Calgary, Alberta, Canada; ^4^Department of Pediatrics, University of Alberta, Edmonton, Alberta, Canada; ^5^Department of Laboratory Medicine and Pathology, University of Alberta, Edmonton, Alberta, Canada; ^6^Alberta Provincial Laboratory for Public Health, Edmonton, Alberta, Canada; ^7^Public Health Ontario Laboratories, University of Toronto, Toronto, Ontario, Canada; ^8^Department of Laboratory Medicine and Pathobiology, University of Toronto, Toronto, Ontario, Canada; ^9^Departments of Pathology & Immunology, Molecular Microbiology, and Pediatrics, Washington University in St. Louis School of Medicine, St. Louis, MO, USA; ^10^Department of Pathology and Laboratory Medicine, BC Children's Hospital, Vancouver, British Columbia, Canada; ^11^University of British Columbia, Vancouver, British Columbia, Canada; ^12^Population, Public and Indigenous Health, Cumming School of Medicine, University of Calgary, Calgary, Alberta, Canada; ^13^Department of Paediatric Laboratory Medicine, The Hospital for Sick Children, Toronto, Ontario, Canada; ^14^Enterics Surveillance and Population Studies Division, Centre for Food-Borne, Environmental and Zoonotic Infectious Diseases, Public Health Agency of Canada, Guelph, Ontario, Canada; ^15^Division of Gastroenterology, Hepatology, and Nutrition, Department of Pediatrics, Washington University School of Medicine, St. Louis, MO, USA; ^16^Sections of Pediatric Emergency Medicine and Gastroenterology, Departments of Pediatrics, University of Calgary, Alberta Children's Hospital, Calgary, Alberta, Canada; ^17^Alberta Children's Hospital Research Institute, Cumming School of Medicine, Calgary, Alberta, Canada

## Abstract

**Background:**

We sought to develop diagnostic test guidance definitions for pediatric enteric infections to facilitate the interpretation of positive test results in the era of multianalyte molecular diagnostic test platforms.

**Methods:**

We employed a systematic, two-phase, modified Delphi consensus process consisting of three web-based surveys and an expert panel face-to-face meeting. In phase 1, we surveyed an advisory panel of North American experts to select pathogens requiring diagnostic test guidance definition development. In phase 2, we convened a 14-member expert panel to develop, refine, and select the final definitions through two web-based questionnaires interspersed with a face-to-face meeting. Both questionnaires asked panelists to rate the degree to which they agreed that if the definition is met the pathogen is likely to be causative of clinical illness.

**Results:**

The advisory panel survey identified 19 pathogens requiring definitions. In the expert panel premeeting survey, 13 of the 19 definitions evaluated were rated as being highly likely (“agree” or “strongly agree”) to be responsible for acute gastroenteritis symptoms by ≥67% of respondent panel members. The definitions for the remaining six pathogens (*Aeromonas, Clostridium difficile, Edwardsiella,* nonenteric adenovirus, astrovirus, and *Entamoeba histolytica*) were indeterminate. After the expert panel meeting, only two of the modified definitions, *C. difficile* and *E. histolytica/dispar*, failed to achieve the *a priori* specified threshold of ≥67% agreement.

**Conclusions:**

We developed diagnostic test guidance definitions to assist healthcare providers for 17 enteric pathogens. We identified two pathogens that require further research and definition development.

## 1. Introduction

Diarrheal disease is the second leading cause of child mortality worldwide [1, 2] and is among the most common causes of illness globally, with over 4 billion episodes occurring annually [3–5]. In the United States, there are 178.8 million episodes of diarrhea contributing to 473,832 hospitalizations each year [6]. Infection by a variety of gastrointestinal pathogens, including parasites, bacteria, and viruses, can cause vomiting and diarrhea that typifies acute gastroenteritis. Multiple diagnostic testing methods are available to identify enteric pathogens including traditional culture techniques for enteric bacteria, direct microscopy for parasites, enzyme immunoassay (EIA) for select enteric viruses, bacterial toxins and some parasites, and molecular methods for specific targets. Despite the availability of multiple modalities to identify enteric pathogens, the etiology often remains unidentified. However, recent practice changes including the collection of rectal swabs rather than stool specimens [7–9] to increase specimen acquisition rates, and the increasing use of molecular methods, especially syndromic panels which detect multiple pathogens simultaneously [10–12] have significantly increased the detection rates of enteric pathogens in symptomatic individuals [13].

Existing guidelines on the diagnosis [14] and management [15] of enteric infectious diseases do not adequately consider the implications of recent advances in sample collection or molecular diagnostic techniques. For example, recently published adult guidelines by the American College of Gastroenterology suggest that the best applicability for molecular diagnostic tests is for the clinician in practice, but do not provide guidance on how clinicians should interpret the results of such testing [16]. The implementation of commercial multiplex gastrointestinal pathogen panels presents practical challenges to healthcare providers in assessing the clinical relevance of positive results and to public health officials who are responsible for surveillance and follow-up of reportable infectious agents. Positive results generated by molecular assays cannot distinguish between asymptomatic carriage, subclinical infection, active disease, resolved disease, or the presence of target nucleic acids liberated by nonpathogens [16]. High rates of enteric pathogen detection have been reported in asymptomatic children [17]. Commercially available multianalyte panels also identify organisms that were not previously routinely tested for such as non-O157 Shiga toxin-producing *Escherichia coli* (STEC). These challenges highlight the need for the use of standardized definitions to guide the interpretation of multiplex polymerase chain reaction (PCR) results by clinicians who often have limited knowledge of laboratory testing procedures. While definitions do exist for some enteric pathogens in the context of public health and outbreak management, no specific definitions exist to guide interpretation and management by healthcare providers. The latter are important because accurate diagnosis by front-line clinicians is crucial to identify those patients who would benefit from antimicrobial therapy and those where unnecessary antibiotics may cause harm [15].

Given the existing knowledge gap, and the paucity of high-quality evidence supporting the clinical interpretation of multianalyte molecular diagnostic test platforms, we conducted a modified Delphi consensus process to develop provisional diagnostic test guidance definitions for pediatric enteric infections to guide the interpretation, by clinicians, of positive diagnostic test results. The goal was to provide guidance for clinicians providing care to children up to 18 years of age with symptoms of acute gastroenteritis defined as a decrease in stool consistency and/or by the presence of three or more episodes of stool or vomiting in a 24-hour period with duration of symptoms of less than seven days [18, 19].

## 2. Materials and Methods

The approach consisted of a systematic, two-phase, modified Delphi consensus methodology with three web-based surveys and an expert panel face-to-face meeting [20]. Ethics approval for this study was obtained by the Conjoint Health Research Ethics Board of the University of Calgary.

### 2.1. Phase 1: Selection of Pathogens Requiring Diagnostic Test Guidance Definition Development

An advisory panel was convened to (1) identify pathogens that require further definition development and (2) select diagnostic tests including culture, electron microscopy (EM), PCR and EIA, and specimen types (i.e., stool, oral, and rectal swabs [21]), which, if positive, would fulfill the definition for each agent. The advisory panel consisted of pediatricians, pediatric emergency medicine and infectious diseases physicians, gastroenterologists, medical microbiologists, and experts in virology and laboratory medicine. Selection criteria for panel members included interest or expertise in pediatric enteric infections as demonstrated by publications in the field, experience in infectious diseases, geographic and practice diversity, and balance between individuals with clinical and laboratory expertise. Candidate panel members received a written invitation to participate that contained a description of study goals, responsibilities, and timelines.

Candidate advisory panel members received a web-based survey managed in REDCap [22] in which they were asked to (1) rate the degree to which they agreed, on a scale of 1-disagree strongly to 5-agree strongly, that a positive result for each of pathogen/diagnostic test/sample collection methods is causative of the child's symptoms. It was determined *a priori* that any pathogen which was unanimously rated as “agree strongly” for all diagnostic test/sample collection modalities would be removed from further consideration as unanimous agreement would indicate that further definition is not required. All other pathogens were retained for further definition development. Free text comments were permitted.

We assessed a list of 19 agents (11 bacteria, 5 viruses, and 3 parasites) that were included in the diagnostic testing panels being evaluated as part of the APPETITE (Alberta Provincial Pediatric EnTeric Infection TEam) project [7]. APPETITE is a provincial initiative designed to comprehensively understand the epidemiology of acute pediatric enteric infections employing rectal swabs and stool samples and a comprehensive diagnostic testing approach.

### 2.2. Phase 2: Development of Diagnostic Test Guidance Definitions

The study team identified existing definitions for each pathogen retained from phase 1 based on a review of Canada's provincial public health case definitions. For pathogens with no existing syndromic-based definitions, the study team developed candidate definitions based on the results of the advisory panel survey in phase 1.

### 2.3. Expert Panel

We convened an expert panel to refine and select the final definitions. The expert panel consisted of individuals trained in pediatrics, infectious diseases, public health, gastroenterology, medical microbiology, virology, and laboratory medicine. Panelists were selected by study investigators from the initial advisory panel and based on recommendations from APPETITE's International Scientific Advisory Committees [7]. Selection criteria included interest or expertise in pediatric enteric infections, clinical decision making experience, geographic and practice diversity, and publications.

A description of research project goals, a summary of the advisory panel survey results, and the existing and newly developed evidence-based definitions were sent to expert panel members. A modified Delphi technique consisting of two rounds of anonymous questionnaires, and a face-to-face meeting was used to generate final study definitions.

#### 2.3.1. Expert Panel Survey 1

The first web-based questionnaire asked panelists to rate the degree to which they agreed that if the definition is met, in a child with vomiting and/or diarrhea, the pathogen is likely to be the cause of the illness. All definitions were specified to be in the context of (1) clinical illness with acute gastroenteritis defined as a decrease in stool consistency and/or by the presence of three or more episodes of loose stool or vomiting in a 24-hour period with a duration of symptoms less than seven days and (2) a positive laboratory diagnostic test for a single pathogen (i.e., no codetection). Specimens considered included stool as well as oral [23] and rectal swabs. Panelists were asked to rate each definition employing a Likert scale ranging from 1 (strongly disagree) to 5 (strongly agree). An option was provided to suggest additional or alternate definitions.

#### 2.3.2. Face-to-Face Meeting

After completion of the first survey, an expert panel face-to-face meeting was convened in Edmonton, Alberta, on March 4, 2016. At the meeting, panelists reviewed anonymized ratings of all the definitions. It was determined *a priori* that unanimously highly rated definitions (i.e., those rated “agree” or “agree strongly” by all respondents) would be retained, and unanimously low rated definitions (i.e., rated “disagree strongly” or “disagree” by all respondents) would be discarded and not discussed further. Panelists were asked to refine highly scored definitions (i.e., ≥67% of panelists rated “agree” or “strongly agree”) and to discuss the remaining definitions.

#### 2.3.3. Expert Panel Survey 2

After the face-to-face meeting, expert panel members received a final survey requesting that they independently re-rate each of the refined definition using the same rating scale. Definitions rated “agree” or “strongly agree” by ≥67% of panelists were retained in the final list of definitions. The threshold for retention was determined *a priori* by the expert panel at the outset of the expert panel meeting and has been previously used with such methods [24]. Pathogens for which a consensus definition could not be reached (i.e., further research required) were identified.

## 3. Results

### 3.1. Phase 1

**Figure 1:**
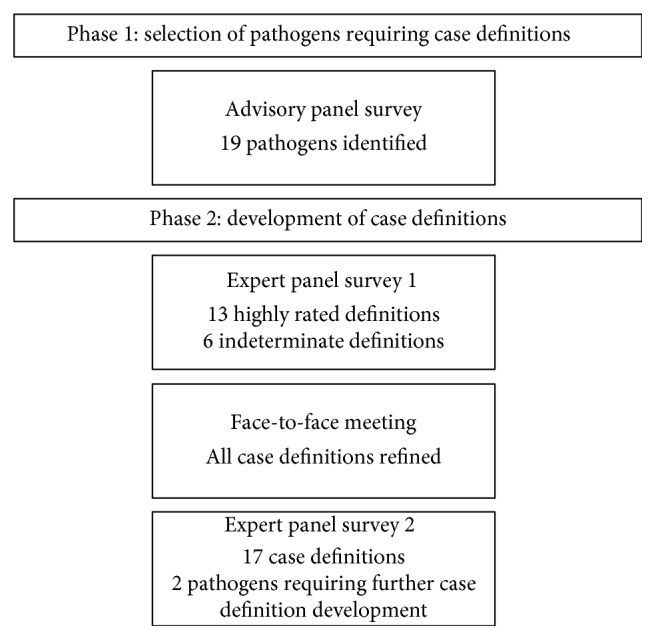
Modified Delphi consensus process.

Modified Delphi consensus process used to develop diagnostic test guidance definitions for pediatric enteric diseases is shown in Figure 1.

The advisory panel web-based survey was initially distributed to 88 individuals on March 26, 2015, with two follow-up e-mail reminders. Thirty-seven of 88 invited experts (42%) completed the advisory panel survey. The majority (89%; 33/37) of respondents were ≥40 years of age and practiced in Canada (76%; 28/37); the remaining 24% (9/37) were from the United States. The majority of the Canadian participants were from Alberta (52%; 14/27). Other provinces and territories represented included British Columbia, Northwest Territories, Nova Scotia, Ontario, and Quebec. Advisory panel participants included individuals with expertise in medical microbiology, pediatric infectious disease, pediatric emergency medicine, community and public health, and laboratory medicine (see Supplemental Table 1). Respondents were similar to nonrespondents with respect to specialty and geographic location.

No pathogen-test method had unanimous agreement (i.e., rated “strongly agree” by all) in terms of assigning causality of symptoms in a child with acute gastroenteritis (Table 1). Consequently, all 19 pathogens were considered to require definition development. For bacterial pathogens, the median proportion agreeing with causality (i.e., rated “agree” or “strongly agree”) was higher for stool culture (90%) compared with stool PCR (83%) and oral swab PCR (30%). The bacterial pathogen with the lowest percent endorsement by both culture (40%; 95% CI: 23, 59) and PCR (30%; 95% CI: 15, 50) was *Edwardsiella*. *Aeromonas* and *Clostridium difficile* also generated <50% agreement as to likelihood of being etiologic when identified. Viral pathogens identified by stool PCR had high levels of agreement as likely being the cause of the symptoms (range: 75–97%) with the exception of general adenovirus which was associated as etiologic by only 51% of respondents (95% CI: 34%, 68%). All positive results on stool evaluated for parasites were endorsed by over 50% of study participants.

### 3.2. Phase 2

#### 3.2.1. Expert Panel: Premeeting Survey

Fourteen of 17 (82%) individuals invited to participate in the in-person expert panel process completed a premeeting definition survey (Supplemental Table 2). The survey was distributed on January 22, 2016, with an e-mail reminder one week later (Table 2). Thirteen of the 19 definitions evaluated were rated as being highly likely (“agree” or “strongly agree”) to be responsible for acute gastroenteritis symptoms by ≥67% of respondent panel members. The definitions for the remaining six agents (*Aeromonas, C. difficile, Edwardsiella,* nonenteric adenovirus, astrovirus, and *Entamoeba histolytica*) were indeterminate. The lowest rated bacterial pathogen was *C. difficile* (36%; 95% CI: 14, 64) while *Escherichia coli* O157 : H7 was the only pathogen classified as etiologic by 100% of respondents. Adenovirus was the lowest rated (36%; 95% CI: 14, 64) viral pathogen while norovirus was the only pathogen classified as etiologic by 100% of respondents.

#### 3.2.2. Expert Panel: Meeting

Twelve of 14 (86%) expert panel members convened at a meeting on March 5, 2016, in Edmonton, Alberta. The discussion focused on achieving definitions which could obtain broad endorsement by experts. The results of the advisory panel survey (Table 1) and the first expert panel survey (Table 2) were presented and used to modify the definitions. The definitions for all 19 pathogens were discussed and revised with the meeting focused primarily on reviewing the six agents with indeterminate ratings. Revisions included the specific wording of both the definition and the agent. For example, the generic term “nucleic acid test” (NAT) was adopted to replace more specific terminology such as PCR. *Yersinia pseudotuberculosis* was removed from the definition for *Yersinia enterocolitica* because the expert panel felt it is unclear if *Yersinia pseudotuberculosis* causes GI illness. For viruses, adenovirus (any serotype) was reworded to adenovirus (nontyped). For parasites, *Entamoeba histolytica* was updated to *Entamoeba histolytica/dispar* because, although dispar is not pathogenic, the two agents cannot be differentiated using direct microscopy or by most antigen-detection tests [25–27]. The definitions that emerged from this process were then recirculated via a final survey to all attendees.

#### 3.2.3. Expert Panel: Postmeeting Survey

All twelve experts who attended the panel meeting completed the final survey which was distributed on March 21, 2016 (100% response rate; Table 3). Eight of the final definitions had a proportional agreement of 100%. *C. difficile* and *E. histolytica/dispar* did not achieve the *a priori* specified threshold of ≥67% agreement. Sixteen of the 19 (84%) modified definitions had a higher proportion agreement than the definitions presented in the expert panel premeeting survey.

## 4. Discussion

This rigorous and systematic process provides expert consensus-based diagnostic test guidance definitions for 17 organisms contained in gastroenteritis nonculture detection panels. These definitions provide guidance to healthcare providers on the interpretation of positive diagnostic test results in the era of multianalyte molecular diagnostics. We identified two pathogens (*C. difficile* and *E. histolytica/dispar*) for which further research and definition development is required.

Our definition work is important as multianalyte enteric pathogen detection approaches are rapidly being adopted across North America. Many healthcare providers may have a limited understanding that these molecular techniques detect nucleic acids of targeted organisms but do not differentiate nonviable organisms or free DNA/RNA from viable pathogens. Positive results generated by molecular assays do not definitively distinguish between asymptomatic carriage, subclinical infection, active disease, resolved disease, or the presence of nucleic acids originating in nonpathogens. They also do not provide definitive information about virulence potential, and asymptomatic children have been found to have high rates of enteric pathogen identification [17, 28].

A positive *C. difficile* test represents the classic challenge clinicians face in multianalyte arrays as its presence may represent colonization and not pathogenic infection, particularly in infancy [11, 13, 28, 29]. Many multianalyte panels also identify organisms that were not previously routinely sought, such as enteroaggregative *E. coli* (EAEC), and enteropathogenic *E. coli* (EPEC). The latter two bacteria have been identified in 84% of mixed infections, raising questions about the clinical significance of their identification and the validity of the definitions employed [13]. As such, the definitions developed in our study can serve as a starting point for clinicians interpreting the results of multianalyte arrays.

Our study also reflects current understanding of the use of oral swab NAT for the diagnosis of bacterial and viral enteric organisms. The paucity of evidence regarding the utility of oral swabs is reflected in the poor ratings for the use of oral swabs in our advisory panel survey and has subsequently been supported by a recent study [21]. As a result, oral swabs are not included in any of the definitions with the exception of norovirus for which there is some evidence of clinical utility [23, 30].

We were unable to develop consensus-based diagnostic test guidance definitions for two pathogens, *C. difficile* and *E. histolytica/dispar*. This is not surprising given the challenges surrounding the accurate diagnosis of *C. difficile*. Among healthy adults, asymptomatic *C. difficile* colonization prevalence varies between 0 [31] and 7% [32], and it is even higher in healthy newborns and infants [33]. The prevalence of *C. difficile* colonization decreases from 35% to 40% during the first month of life to approximately 15% by one year of age [34]. However, 10–20% of older infants continue to asymptomatically carry toxigenic organisms and the associated toxin genes, and in many asymptomatic children, the toxins are well expressed [28, 35]. In addition, there is limited evidence on the duration of the colonized state, the risk factors related to transmission, and the mechanisms underlying progression from colonization to disease [33]. The lack of a standardized definition for asymptomatic colonization, or carriage, makes the interpretation of test findings, and the development of a diagnostic test guidance definition, difficult. Further research is required to develop an evidence based, standardized definition for *C. difficile* disease, and equally importantly, colonization.

In the developing world, *E. histolytica* is a common cause of protozoan morbidity and mortality [36]. However, infection is uncommon in North America, and occurs primarily in immigrants and travelers from developing countries [37]. Nonetheless, asymptomatic colonization is common, occurring in up to 4% of individuals in high-risk areas [38], such as California, and it occurs with both *E. histolytica* and the nonpathogenic *E. dispar*. Colonization with the morphologically identical parasite *E. dispar* is three times more common in developing countries and at least ten times more common in developed nations [37]. Routine diagnostic tests such as direct microscopy cannot differentiate between *E. histolytica* and *E. dispar* based on morphologic criteria, so most laboratory reports indicate *E. histolytica/dispar* [14]. The relatively low prevalence in North America, combined with the high colonization rates with both the pathogenic, and nonpathogenic form, and the difficulty in differentiating the two, likely account for the lack of consensus for a diagnostic test guidance definition. It should also be noted that the panel consisted of individuals who have largely practiced in North America, where amebiasis is exceptionally rare in childhood. However, recently published work on the most common causes of diarrhea using molecular methods indicated that even in Asia and Africa, the most common causes of diarrhea were *Shigella*, rotavirus, adenovirus 40/41, heat-stable enterotoxin-producing *E. coli, Cryptosporidium*, and *Campylobacter*, which supports the broad-based utility of our clinical definitions [2].

A strength of this project is the use of a rigorous consensus methodology consisting of multiple survey rounds and a face-to-face meeting, which enabled dialogue [20]. Similar methods have been used to combine best evidence and expert opinion in a number of clinical and research settings [39, 40] including antimicrobial stewardship [41]. Both our advisory and expert panels had geographic representation from throughout North America and including a diversity of disciplines, thereby contributing to the utility, credibility, and generalizability of our definitions. We had representation from a broad spectrum of stakeholders involved in the diagnosis and management of pediatric infectious enteric diseases including front-line clinicians, medical microbiologists, infectious disease specialists, and public health experts. The engagement of our expert panel, with 86% of our expert panel participating in the face-to-face meeting, and a 100% response rate on our final survey, is evidence of the relevance and importance of the topic and the validity of the results. Nonetheless, our definitions can be considered as a derivation set; future work should include a validation with a distinct expert panel.

It should be noted that the definitions provided are only applicable in the setting of a test that is positive for a single pathogen. Clinicians should also be cognizant that in most cases where the definitions provided are met, therapy remains predominantly supportive and few of the enteropathogens included in Table 3 require antimicrobial treatment, which can at times even be detrimental [42]. Although multiple pathogens are detected in ∼15% of stool specimens in children with diarrhea [10], determining guidelines for the interpretation of a positive test for more than one pathogen was beyond the scope of this project. The guidance provided is also very general and does not incorporate the myriad of clinical scenarios that can occur. The definitions are not intended to replace expert opinion (e.g., infectious disease specialist, medical microbiologist) but rather are intended to assist clinicians in the context of otherwise healthy children with typical clinical features and <7 days of symptoms. We also identified two agents, *C. difficile* and *E. histolytica/dispar*, for which further research is required to develop accurate diagnostic test guidance definitions. Prospective research currently underway [7] is attempting to validate these definitions in the clinical setting of pediatric enteric illness.

We also did not assess the confidence in the individual items included in each definition, nor the potential role of quantitative molecular techniques [2]. Thus, although the definitions may include multiple diagnostic testing methods, the test characteristics of each modality vary. While these definitions may lead clinicians to conclude that all diagnostic tests are created equal, as diagnostic testing evolves and progresses, less accurate tests are phased out and replaced by superior diagnostic tests. It should also be noted that our process did not include some of the diarrheagenic *E. coli* strains (e.g., ETEC, EAEC, and EPEC) that are included in some multianalyte tests while it did include some pathogens not routinely identified by all laboratories (e.g., *Edwardsiella*). This likely stems from the fact that few clinicians and microbiologists have any significant experience with the detection of these enteric pathogens outside of research settings. Currently, very few commercially available assays include all these pathogen targets [43]. As experience is accumulated with the detection of these pathogens in clinical laboratories, it would certainly be useful to include them in a subsequent evaluation. Finally, as we had to limit the number of study participants, and their expert opinions may not reflect the entire spectrum of expertise, our findings should not be interpreted as the only perspective on this topic.

In conclusion, we report a rigorous, systematic, expert consensus process that includes diagnostic test guidance definitions for 17 enteric organisms. These definitions can be employed by healthcare providers to interpret positive diagnostic test results in the era of multianalyte molecular diagnostics as we await further clinical data, particularly with respect to consideration of clinical settings and interpretation of detection of more than one pathogen. We also identified pathogens for which further definition development is required.

## Figures and Tables

**Table 1:** Results from initial advisory panel survey reporting respondents who rated “agree” or “strongly agree” that the pathogen/test/collection modality was causative of symptoms in a child with acute gastroenteritis.

Pathogen diagnostic test	Proportion agreement *N* (%)	95% confidence interval
Bacterial pathogens		
*Aeromonas*		
Stool culture	43	26, 62
Stool PCR	37	21, 56
Oral swab PCR	13	4, 32
*Campylobacter*		
Stool culture	97	81, 100
Stool PCR	83	65, 94
Oral swab PCR	30	15, 50
*Clostridium difficile* antigen		
Stool EIA	50	32, 68
Stool PCR	47	29, 65
Oral swab PCR	17	6, 35
*Edwardsiella*		
Stool culture	40	23, 59
Stool PCR	30	15, 50
Oral swab PCR	17	6, 35
*Escherichia coli* O157		
Stool culture	97	81, 100
Stool PCR	87	68, 96
Oral swab PCR	37	21, 56
Enterotoxigenic *Escherichia coli* (ETEC)		
Stool culture	90	73, 97
Stool PCR	84	66, 94
Oral swab PCR	29	15, 48
*Salmonella* spp.		
Stool culture	81	62, 92
Stool PCR	77	57, 89
Oral swab PCR	20	8, 39
*Shigella* spp.		
Stool culture	97	81, 100
Stool PCR	90	74, 97
Oral swab PCR	33	18, 53
Shiga toxin-producing *Escherichia coli* (STEC)		
Stool culture	87	68, 96
Stool PCR	80	61, 92
Oral swab PCR	33	18, 53
*Vibrio cholera*		
Stool culture	93	76, 99
Stool PCR	83	65, 94
Oral swab PCR	33	18, 53
*Yersinia enterocolitica*		
Stool culture	93	76, 99
Stool PCR	83	65, 94
Oral swab PCR	33	18, 53

Viral pathogens		
Adenovirus (any serotype)		
Stool PCR	51	34, 68
Oral swab PCR	31	17, 49
Adenovirus 40/41		
Stool PCR	81	63, 91
Oral swab PCR	54	37, 71
Astrovirus		
Stool PCR	83	66, 93
Oral swab PCR	47	30, 65
Norovirus GI/GII		
Stool PCR	94	80, 99
Oral swab PCR	60	42, 76
Rotavirus		
Stool PCR	97	84, 100
Oral swab PCR	43	28, 60
Sapovirus		
Stool PCR	75	57, 87
Oral swab PCR	40	24, 58

Parasitic pathogens		
*Cryptosporidium* spp.		
Stool ova and parasite	86	66, 95
Stool PCR	68	48, 83
Stool EIA	64	44, 81
*Entamoeba histolytica*		
Stool ova and parasite	57	37, 75
Stool culture	68	48, 83
Stool PCR	61	41, 78
Stool EIA	75	55, 89
*Giardia lamblia*		
Stool ova and parasite	86	66, 95
Stool PCR	68	48, 83
Stool EIA	75	55, 89

EIA, enzyme immunoassay; PCR, polymerase chain reaction. Stool culture, ova and parasite, and EIA: bulk stool and/or rectal swab.

**Table 2:** Expert panel survey, prior to in-person meeting, where attendees rated the degree to which they agreed that if the provided definition was met, in a child with vomiting and/or diarrhea, the pathogen is highly likely to be causative of clinical illness.

Pathogen	Definition	Strongly disagree	Disagree	Neither agree nor disagree	Agree	Strongly agree	Proportion agreement (%)
Bacterial pathogens, *N* (%)							
*Aeromonas*	Isolation of *Aeromonas* in a stool specimen via culture or PCR	0	0	2/14 (14%)	9/14 (64%)	3/14 (21%)	85
*Campylobacter*	Isolation of *Campylobacter* spp. from a stool specimen via culture or PCR	0	0	4/14 (29%)	6/14 (43%)	4/14 (29%)	72
*Clostridium difficile*	A positive toxin A or B assay for *Clostridium difficile* from a stool specimen via PCR for toxin A/B	1/14 (7%)	4/14 (29%)	4/14 (29%)	2/14 (14%)	(3/14) (21%)	36
*Edwardsiella*	Isolation of *Edwardsiella* in a stool specimen via culture or PCR	1/14 (7%)	0	7/14 (50%)	6/14 (43%)	0	43
*Escherichia coli* O157 : H7	Isolation of *Escherichia coli* O157 : H7 from a stool specimen via culture, EIA, or PCR	0	0	0	4/14 (29%)	10/14 (71%)	100
*Escherichia coli* (non-O157 : H7)	Isolation of Shiga toxin-producing *Escherichia coli* (non-O157 : H7) from a stool specimen via culture, EIA, or PCR	0	0	1/14 (7%)	8/14 (57%)	5/14 (36%)	93
*Salmonella*	Isolation of spp. from a stool specimen via culture or PCR	0	0	4/13 (31%)	6/13 (46%)	3/13 (23%)	69
*Shigella*	Isolation of *Shigella* spp. from a stool specimen via culture or PCR	0	0	1/13 (8%)	8/13 (62%)	4/13 (31%)	93
*Vibrio cholerae* serotype O1 or O139	Isolation of cholera toxin-producing *Vibrio cholerae* serotype O1 or O139 from a stool specimen via culture or PCR	0	0	2/13 (15%)	4/13 (31%)	7/13 (54%)	85
*Yersinia enterocolitica*	Isolation of *Yersinia enterocolitica* or *Yersinia pseudotuberculosis* from a stool specimen via culture or PCR	0	1/14 (7%)	2/14 (14%)	8/14 (57%)	3/14 (21%)	79

Viral pathogens, *N* (%)							
Rotavirus	Isolation of rotavirus from stool specimen by EM, EIA, latex agglutination, or PCR	0	0	0	8/14 (57%)	6/14 (43%)	100
Adenovirus (any serotype)	Isolation of adenovirus in a stool specimen via PCR	0	2/14 (14%)	7/14 (50%)	5/14 (36%)	0	36
Adenovirus 40/41	Isolation of adenovirus 40/41 in a stool specimen via PCR	0	0	2/14 (14%)	11/14 (79%)	1/14 (7%)	86
Norovirus	Isolation of norovirus from a stool specimen or ORAL SWAB via PCR	0	0	0	(8/14) (57%)	6/14 (43%)	100
Sapovirus	Isolation of sapovirus in a stool specimen via PCR	0	0	2/14 (14%)	9/14 (64%)	3/14 (21%)	85

Parasitic pathogens, *N* (%)							
*Cryptosporidium*	Isolation of *Cryptosporidium* from a stool specimen via microscopy, EIA, or PCR	0	1/14 (7%)	2/14 (14%)	7/14 (50%)	4/14 (29%)	79
*Entamoeba histolytica*	Detection of *Entamoeba histolytica* from a stool specimen via microscopy	1/14 (7%)	1/14 (7%)	4/14 (29%)	4/14 (29%)	4/14 (29%)	58
*Giardia lamblia*	Detection of *Giardia lamblia* in a stool specimen via microscopy or EIA	0	1/14 (7%)	1/14 (7%)	10/14 (71%)	2/14 (14%)	85

EIA, enzyme immunoassay; PCR, polymerase chain reaction. Stool specimen includes bulk stool and/or rectal swab. All definitions are in the context of a single-positive pathogen.

**Table 3:** Definitions that emerged from in-person meeting and subsequently circulated for final evaluation by the definition meeting attendees.

Pathogen	Case definition	Strongly disagree	Disagree	Neither agree nor disagree	Agree	Strongly agree	Proportion agreement (%)	Dropped diagnostics from initial (1) and second survey (2)
Bacterial pathogens, *N* (%)								
*Aeromonas*	Detection of *Aeromonas* in a stool specimen via culture or NAT (e.g., PCR)	0	0	3/12 (25%)	8/12 (67%)	1/12 (8%)	75	Oral swab PCR (1); none (2)
*Campylobacter*	Detection of *Campylobacter* spp. from a stool specimen via culture or NAT (e.g., PCR)	0	0	0	8/12 (67%)	4/12 (33%)	100	Oral swab PCR (1); none (2)
*Clostridium difficile*	Detection of *Clostridium difficile* toxin A or B from a stool specimen via NAT (e.g., PCR) for toxin A/B	0	2/12 (17%)	5/12 (42%)	5/12 (42%)	0	42	Stool EIA, oral swab PCR (1); none (2)
*Edwardsiella*	Detection of *Edwardsiella* in a stool specimen via culture	0	0	3/12 (25%)	8/12 (67%)	1/12 (8%)	75	Stool PCR, oral swab PCR (1); none (2)
*Escherichia coli* O157 : H7	Detection of *Escherichia coli* O157 : H7 from a stool specimen via culture, EIA or NAT (e.g., PCR)	0	0	0	5/12 (42%)	7/12 (58%)	100	Oral swab PCR (1); none (2)
*Escherichia coli* (non-O157 : H7)	Detection of shiga toxin-producing *Escherichia coli* (non-O157 : H7) from a stool specimen via culture, EIA or NAT (e.g., PCR)	0	0	0	7/12 (58%)	5/12 (42%)	100	Oral swab PCR (1); none (2)
*Salmonella*	Detection of *Salmonella* spp. from a stool specimen via culture or NAT (e.g., PCR)	0	0	0	7/12 (58%)	5/12 (42%)	100	Oral swab PCR (1); none (2)
*Shigella*	Detection of *Shigella* spp. from a stool specimen via culture or NAT (e.g., PCR)	0	0	0	6/12 (50%)	6/12 (50%)	100	Oral swab PCR (1); none (2)
*Vibrio cholerae* serotype O1 or O139	Detection of *Vibrio cholerae* serotype O1 or O139 from a stool specimen via culture or NAT (e.g., PCR)	0	0	1/12 (8%)	5/12 (42%)	6/12 (50%)	92	Oral swab PCR (1); none (2)
*Yersinia enterocolitica*	Detection of *Yersinia enterocolitica* from a stool specimen via culture or NAT (e.g., PCR)	0	0	0	6/12 (50%)	6/12 (50%)	100	Oral swab PCR (1); none (2)

Viral pathogens, *N* (%)								
Adenovirus (nontyped)	Detection of adenovirus (nontyped) in a stool specimen by EM, EIA, or NAT (e.g., PCR)	1/12 (8%)	1/12 (8%)	1/12 (8%)	8/12 (67%)	1/12 (8%)	75	Oral swab PCR (1); none (2)
Adenovirus 40/41	Detection of adenovirus 40/41 in a stool specimen via EIA or NAT (e.g., PCR)	0	0	1/12 (8%)	6/12 (50%)	5/12 (42%)	92	Oral swab PCR (1); none (2)
Astrovirus	Detection of astrovirus in a stool specimen via NAT (e.g., PCR)	0	1/12 (8%)	1/12 (8%)	8/12 (67%)	2/12 (17%)	84	Oral swab PCR (1); none (2)
Norovirus	Detection of norovirus from a stool specimen or ORAL SWAB via NAT (e.g., PCR)	0	0	0	8/12 (67%)	4/12 (33%)	100	None (1 and 2)
Rotavirus	Detection of rotavirus from a stool specimen by EM, EIA or NAT (e.g., PCR)	0	0	0	4/12 (33%)	8/12 (67%)	100	Oral swab PCR (1); latex agglutination (2)
Sapovirus	Detection of sapovirus in a stool specimen via NAT (e.g., PCR)	0	0	1/12 (8%)	8/12 (67%)	3/12 (25%)	92	Oral swab PCR (1); none (2)
Parasitic pathogens, *N* (%)								
*Cryptosporidium*	Detection of *Cryptosporidium* from a stool specimen via microscopy, EIA or NAT (e.g., PCR)	0	0	1/12 (8%)	6/12 (50%)	5/12 (42%)	92	Stool ova and parasite, oral swab PCR (1); none (2)
*Entamoeba histolytica/dispar*	Detection of *Entamoeba histolytica/dispar* from a stool specimen via microscopy	0	1/12 (8%)	7/12 (58%)	4/12 (33%)	0	33	Stool ova and parasite, stool culture, PCR, and EIA (1); none (2)
*Giardia lamblia*	Detection of *Giardia lamblia* in a stool specimen via microscopy or EIA	0	0	1/12 (8%)	8/12 (67%)	3/12 (25%)	92	Stool ova and parasite, PCR (1); none (2)

EIA, enzyme immunoassay; EM, electron microscopy; NAT, nucleic acid test, PCR, polymerase chain reaction. Stool specimen includes bulk stool and/or rectal swab. All definitions are in the context of a single-positive pathogen.

## Data Availability

All data generated or analysed during this study are included in this published article and the supplemental material.

## Abbreviations

EAEC: Enteroaggregative *E. coli*
EIA: Enzyme immunoassay
EPEC: Enteropathogenic *E. coli*
NAT: Nucleic acid test
PCR: Polymerase chain reaction
STEC: Shiga toxin-producing *Escherichia coli*.

## Supplementary Materials

Supplementary Materials Supplemental Table 1: demographic information for participants of the initial survey. Supplemental Table 2: expert panel members.
