# Supplementary material for: Diagnostic Interpretation Guidance for Pediatric Enteric Pathogens: A Modified Delphi Consensus Process
Source: Can J Infect Dis Med Microbiol. 2018 Sep 27;2018:2589826. doi: 10.1155/2018/2589826 (PMC6180965; doi:10.1155/2018/2589826)
Supplement: Supplementary Materials — Supplemental Table 1: demographic information for participants of the initial survey. Supplemental Table 2: expert panel members. [file 2589826.f1.pdf]

Supplemental Table 1: Demographic Information for Participants of Initial Survey

Characteristic	Numerator	Denominator	Proportion (%)
**Age**			
30 to <40	4	37	10.81
40 to <50	19	37	51.35
50 to <60	8	37	21.62
60 to <70	6	37	16.22
**Country**			
Canada	28	37	75.68
United States	9	37	24.32
**Province**			
Alberta	14	27	51.85
British Columbia	1	27	3.70
Northwest Territories	1	27	3.70
Nova Scotia	2	27	7.41
Ontario	8	27	29.63
Quebec	1	27	3.70
**Area or Expertise/Practice**			
Medical microbiology	14	37	**
Emergency medicine – paediatrics	7	37	**
Infectious disease – paediatrics	7	37	**
Community/public health	6	37	**
Laboratory medicine	6	37	**
Epidemiology	5	37	**
Infectious disease- adult	4	37	**
Gastroenterology – paediatrics	4	37	**
Family medicine	2	37	**
Health economics	1	37	**
Emergency medicine – general	1	37	**
Other	3	37	**

Supplemental Table 2: Expert Panel Members*

Name	City, State/ Province, Country	Academic Affiliation (i.e. university)	Agency Affiliation
Astrid Petrich	Toronto, ON, Canada	University of Toronto	The Hospital for Sick Children
Bonita Lee	Edmonton, AB, Canada	University of Alberta	Alberta Health Services
Brendon Parsons	Edmonton, AB, Canada	University of Alberta	ProvLab Alberta
Carey-Ann Burnham	St. Louis, MO, United States	Washington University	Barnes-Jewish Hospital
David Goldfarb	Hamilton, ON, Canada	University of British Columbia	BC Children`s Hospital
Frank Pollari	Guelph, ON, Canada		Public Health Agency of Canada
Graham Tipples	Edmonton, AB, Canada	University of Alberta	ProvLab Alberta
Judy MacDonald	Calgary, AB, Canada	University of Calgary	Alberta Health Services
Xiao-Li Pang	Edmonton, AB, Canada	University of Alberta	ProvLab Alberta
Linda Chui	Edmonton, AB, Canada	University of Alberta	ProvLab Alberta
Phil Tarr	St. Louis, MO, United States	Washington University	St. Louis Children's Hospital
Ran Zhuo	Edmonton, AB, Canada	University of Alberta	ProvLab Alberta
Stephen Freedman	Calgary, AB, Canada	University of Calgary	Alberta Health Services
Vanessa Allen	Toronto, ON, Canada	University of Toronto	Public Health Ontario

*All participants consented to the publication of their identities.
